# *Pseudomonas koreensis* Recovered From Raw Yak Milk Synthesizes a β-Carboline Derivative With Antimicrobial Properties

**DOI:** 10.3389/fmicb.2019.01728

**Published:** 2019-07-29

**Authors:** Manpreet Kaur, Manoj Jangra, Harjodh Singh, Rushikesh Tambat, Nittu Singh, Sanjay M. Jachak, Sunita Mishra, Charu Sharma, Hemraj Nandanwar, Anil Kumar Pinnaka

**Affiliations:** ^1^Clinical Microbiology and Bioactive Screening Laboratory, Council of Scientific and Industrial Research-Institute of Microbial Technology, Chandigarh, India; ^2^Microbial Type Culture Collection and Gene Bank, Council of Scientific and Industrial Research-Institute of Microbial Technology, Chandigarh, India; ^3^Council of Scientific and Industrial Research-Central Scientific Instruments Organisation, Chandigarh, India; ^4^Academy of Scientific and Innovative Research, Council of Scientific and Industrial Research, New Delhi, India; ^5^Council of Scientific and Industrial Research-Institute of Microbial Technology, Chandigarh, India; ^6^Department of Natural Products, National Institute of Pharmaceutical Education and Research, Sahibzada Ajit Singh Nagar, India

**Keywords:** antibiotic- resistance, natural product, yak milk, antimicrobial compound, β-Carboline

## Abstract

Natural evolution in microbes exposed to antibiotics causes inevitable selection of resistant mutants. This turns out to be a vicious cycle which requires the continuous discovery of new and effective antibiotics. For the last six decades, we have been relying on semisynthetic derivatives of natural products discovered in “Golden Era” from microbes, especially *Streptomyces* sp. Low success rates of rational drug-design sparked a resurgence in the invention of novel natural products or scaffolds from untapped or uncommon microbial niches. Therefore, in this study, we examined the microbial diversity inhabiting the yak milk for their ability to produce antimicrobial compounds. We prepared the crude fermentation extracts of fifty isolates from yak milk and screened them against indicator strains for the inhibitory activity. Later, with the aid of gel filtration chromatography followed by reversed-phase HPLC, we isolated one antimicrobial compound Y5-P1 from the strain Y5 (*Pseudomonas koreensis*) which showed bioactivity against Gram-positive and Gram-negative bacteria. The compound was chemically characterized using HRMS, FTIR, and NMR spectroscopy and identified as 1-acetyl-9H-β-carboline-3-carboxylic acid. It showed minimum inhibitory activity (MIC) in the range of 62.5–250 μg /ml. The cytotoxicity results revealed that IC_50_ against two mammalian cell lines i.e., HepG2 and HEK293T was 500 and 750 μg/ml, respectively. This is the first report on the production of this derivative of β-carboline by the microorganism. Also, the study enlightens the importance of microbes residing in uncommon environments or unexplored habitats in the discovery of a diverse array of natural products which could be designed further as drug candidates against highly resistant pathogens.

## Introduction

The growing burden of antibiotic-resistance has instigated the researchers worldwide to search for the new antimicrobial compounds. Especially talking about bacterial infections, they are responsible for the loss of several thousand lives (expected to be more than cancer or cardiac diseases by 2050) and trillions of money across the globe (O'neill, 2016). In Europe, antibiotic-resistant infections cost around $1.5 billion per annum while in the U.S., the estimated cost is $55 billion as per reported by Center for Disease Control and Prevention (Centers for Disease Control and Prevention, 2014; Bowen, 2017). Moreover, antibiotic-resistance challenges our modern medical procedures such as chemotherapy, organ transplantation etc. Hence antibiotic development against hard-to-deal-with bacteria is a crucial arena of study for healthcare researchers (Thaker et al., 2013). Many pharmaceutical companies developed several molecules using rational drug-designing but the success rate of approval of new antibiotic has been very low and this approach has been unable to tackle the perfect storm of antibiotic-resistance (Andersson and Hughes, 2010; Bush et al., 2011). This situation arises as a global challenge and therefore, we must find an alternate solution to address this issue.

Secondary metabolites produced by untapped microorganisms represent a hidden treasure which includes the new antibiotics and other bioactive molecules (Clardy et al., 2006; Bowen, 2017). These natural products hold great potential to fight pathogenic bacteria (Challinor and Bode, 2015) as is evident from the fact that majority of the antibiotics used in our current drug regimen were originated from microbial sources in the “Golden Antibiotic Era” (Gelband et al., 2015; Giorgi, 2016). Owing to the failure of synthetic chemistry approaches and re-discovery of already available antibiotics, it is, therefore, necessary to exploit the unique and underexplored microbial sources for new scaffolds or chemical compounds. Very recently, few reports have been published on the discovery of novel antibiotics from unculturable bacteria or bacteria from unusual niches (Ling et al., 2015; Zipperer et al., 2016; Chellat and Riedl, 2017; Hover et al., 2018).

In the present study, we selected one of the unique sources i.e., yak milk which is a very unfamiliar consumable form of milk as yaks (*Bos grunniens*) dwell in the high altitude geographical locations, especially in Tibetan plateau in China and Himalayan regions in India which are situated at 3,000 m above the sea level (An et al., 2005; Yang et al., 2010; Bao et al., 2012). The yak milk is highly nutritious in terms of fats, proteins, and minerals when compared with other mammal's milk, and thus helps maintain the health of the native people living in such harsh conditions (Luming et al., 2008; Sangma, 2010; Liu et al., 2011). Previously, we studied the probiotic properties of lactic acid bacteria present in yak milk and found that most of the strains showed *in vitro* health benefits including the inhibition of growth of pathogenic bacteria (Kaur et al., 2017). These results encouraged us to further examine its microbial diversity for antibiotic production. The present work demonstrates the antimicrobial properties of one isolate Y5, identified as *Pseudomonas koreensis (P. koreensis)*. We isolated and characterized one β-carboline derivative (Y5-P1) from this strain. We also studied its antimicrobial activity in drug-resistant pathogens and performed its mammalian cytotoxicity. To the best of our knowledge, this is the first report on the production of this derivative from a microorganism, and also *P. koreensis* is not reported previously for producing an antimicrobial compound. This study also highlights that the microflora from rare habitats such as yak milk could be a source to screen for antibiotic compounds.

## Methodology

### Isolation and Screening of Bacterial Isolates for Antimicrobial Activity

Fresh yak milk was purchased from the local market near Chang La (34°03′32″N77°55′51″E) in the Ladakh region, India. The milk sample was transferred to sterile tubes and brought to laboratory at 4°C conditions. Afterwards, the sample was serially diluted in phosphate buffered saline (PBS) and spread plated on different media plates to isolate the microbial diversity present. The bacteria were isolated and screened for antimicrobial activity. In brief, all the isolates were grown in 50 ml of tryptic soy broth at 30°C. Post 24 or 48 h incubation, the cell-free supernatant (CFS) was taken by centrifugation. At 48 h the crude extract was prepared using activated Diaion HP-20 resins (Sigma-Aldrich). Antimicrobial activity of CFS and extracts was measured with agar well-diffusion assay according to the CLSI guidelines. The activity was assayed against *Candida albicans* MTCC 224, *Staphylococcus aureus* ATCC 25923, *Escherichia coli* MTCC 1610, *Klebsiella pneumoniae* MTCC 618, *Bacillus subtilis* ATCC 6633, *Micrococcus luteus* MTCC 106, *Listeria monocytogenes* MTCC 839, *Vibrio cholera* MTCC 3904 and *Pseudomonas aeruginosa* MTCC 1934. Those isolates showing consistent antimicrobial activity and previously unreported for antibiotic production were selected.

### Antimicrobial Producing Strain and Growth Curve Analysis

All the positive strains were identified using 16S rRNA gene sequencing. Strain Y-5, which showed better and consistent activity against Gram-positive and Gram-negative bacteria, was chosen for the purification of the antimicrobial compound since this bacterium was previously unreported for its inhibitory activity. To determine the growth pattern with respect to inhibitory activity, the strain Y-5 was inoculated in the nutrient broth and incubated at 30°C. One ml of culture was taken at different time intervals, and the optical density (OD_600_) was recorded using Shimadzu UV-Visible spectrophotometer. Bioactivity of the CFS at each time point was also tested using agar well-diffusion assay against the indicator strain i.e., *B. subtilis*. Antimicrobial activity was noted as a zone of inhibition in mm. A graph between time vs. optical density and antimicrobial activity was plotted.

### Fermentation of Strain Y5 and Crude Extract Preparation

Primary culture (inoculum) was prepared by inoculating one colony of Y5-strain in 50 ml of NB medium in a 100 ml flask from a fresh culture plate and keeping overnight at 30°C. Ten milliliters of the primary culture was used to inoculate the 1L NB medium in 2L flask and fermentation was carried out at 30°C for 48 h at 180 rpm. The culture was harvested by centrifugation (10,000 × g) for 15 min, and CFS was used to prepare crude extract using Diaion HP-20 resin and the cell biomass was discarded. Briefly, activated resins at a concentration of 10% (v/v) were mixed with the CFS and incubated for 2–3 h to allow the binding of the antimicrobial compounds to the resins. Afterward, the Diaion beads were filtered and washed with water to remove unbound media components or impurities. The compounds adsorbed on the resins were eluted successively with a step gradient of 70% and 100% methanol: isopropanol: acetone (6:3:1) with 1 h incubation at each step. The fractions were then evaporated using rotary evaporator (BUCHI, R-300) at 45°C and reconstituted in Milli-Q water to make the final volume 50–60 ml (methanol was added in later fraction to dissolve the extract completely). The fermentation was carried out in batches of 6-liter (six flasks containing 1 L medium each), and after extraction, the extracts were stored at 4°C for further purification steps. Antimicrobial activity of the crude extract was assayed.

### Purification of Antibacterial Compound

The active fractions (filtered through 0.45 μm membrane filter) of Diaion-HP 20 were loaded on Sephadex LH-20 column (GE- Healthcare, USA). The column (50 cm × 29 mm) was pre-equilibrated with two column volume of water (column volume ~300 ml). Twenty to twenty five milliliters of sample was loaded and sequentially eluted with step gradients of water and methanol (0, 10, 20, and 30–100%) with 100 ml each. Absolute methanol was run until all the compounds eluted thoroughly. Fractions (40 ml each) were collected and concentrated using Rotavap up to 2 to 3 ml for bioactivity assay. Active fractions were pooled based on their activity pattern and were purified on Reversed-Phase High-Performance Liquid Chromatography (RP-HPLC) using Agilent prep HPLC system. C4 column (Phenomenex, 10 × 250 mm, 5 μm) was employed with water plus 10% acetonitrile as mobile phase A and 100% acetonitrile as mobile phase B. During the initial HPLC methods development, all fractions, and separable peaks were assessed for bioactivity until the final method for purification of the single active peak was established. Following gradient was used for the separation of active compound from LH-20 active fraction: 2% solvent B to 23% solvent B over 13 min, and 23% B to 75% B in 8 min, and reverse gradient of 75% to 2% in 5 min. The flow rate was kept constant at 5.0 ml/min and 7 min time was given for equilibration before the next injection. The active peak (RT 10.5 min) was collected and concentrated for activity assay. This compound was named as Y5-P1, and multiple injections were performed to generate the sufficient quantity of the compound. The purity of peak was analyzed by the analytical HPLC (Shimadzu UFLC with LC-20AD pump and PDA detector) using the same column and the solvent system. The purified compound was lyophilized further to get yellow colored powder. One batch of 6 liter broth yielded 10–15 milligrams of the pure compound. This compound was used for the structural and biological characterization.

### Mass Spectroscopy and FTIR Analysis

Y5-P1 was subjected to LC-ESI-MS (Agilent 6550i funnel Q-TOF) and ESI-HR-MS (Waters). The compound precursor ions were detected in both positive and negative ion mode with scan range m/z 50 to m/z 1,500. For the Fourier transform infrared (FTIR) analysis, the sample was prepared by grinding finely one milligram of the compound with ninety-five milligrams of potassium bromide to make thin film. Overnight desiccation was performed to remove any moisture present in the sample. The FTIR spectrum was recorded on Perkin Elmer spectrophotometer in the range of 4,000–400 cm^−1^.

### Nucleic Magnetic Resonance (NMR) Spectroscopy

For NMR analysis, ~12 mg of the compound was dissolved in 600 μl of deuterated dimethyl sulfoxide (DMSO-d_6_). All the NMR spectra were acquired on Bruker (400MHz) spectrometer. ^1^H NMR and ^13^C NMR spectra were referenced to the internal standard tetramethylsilane, in a deuterated solvent. Coupling constants (J) were reported in Hertz. All assignments were verified using two-dimensional ^1^H-^1^H (COSY), ^1^H-^13^C (HSQC), and HMBC experiments.

### Minimum Inhibitory Concentration (MIC) Determination

Minimum inhibitory concentration (MIC) of the purified compound was determined against the standard strains and clinical isolates were determined using 96-well microtiter plate dilution method as per CLSI Guidelines. Test strains were grown in MHB (Mueller Hinton Broth) at 37°C, 180 rpm. The culture was grown until mid-exponential growth phase with an OD_600_ of 0.4. The culture was adjusted to the final bacterial count of ~4 × 10^5^ CFU/ml. Hundred milliliters of sterilized media containing the 2-fold serial dilutions of the compound were added to each well. Then, 100 μl of prepared culture was added and the plate was incubated at 37°C for 18 h. The lowest concentration with no visible growth in the well was considered as the MIC.

### Time-Dependent Killing Kinetics

*Staphylococcus aureus* ATCC 25923 cells were adjusted to 4 × 10^5^ CFU/ml in CA-MHB and treated with two different concentrations of Y5-P1, i.e., 0.5 × MIC and 1 × MIC. At different time intervals, the sample was taken and spread plated on fresh MHA plates to determine the number of colonies appeared. The sample without antimicrobial compound served as positive control. The experiment was performed in triplicate and the data were plotted as number of CFU/ml vs. time.

### Transmission Electron Microscopy

*Staphylococcus aureus* cells (~10^7^ CFU/ml) were treated in one milliliter CA-MHB with Y5-P1 at a concentration of its MIC for 2 h at 37°C. Post incubation, the excess compound was removed by centrifugation and the cells were resuspended in 100 μl of PBS. Untreated cells were processed in similar manner. The cells were fixed in 2.5% glutaraldehyde in PBS and placed on carbon coated copper grids (Polysciences, cat# 24933). The cells were negatively stained with phosphotungstic acid (PTA) to eliminate the interference from background. The specimens were observed under electron microscope (JEOL, JEM-2100) at 200 kV.

### Mammalian Cytotoxicity

3-(4, 5-Dimethylthiazol-2-Yl)-2, 5-Diphenyltetrazolium Bromide (MTT) assay was used to determine the cytotoxicity of compound Y5-P1 on HEK-293T and HepG2 cell lines. HEK- 293T and HepG2 cells were seeded in triplicates in 96-well plate and incubated for 24 h at 37°C, 5% CO_2_. After 24 h, the compound was added to the cells at different concentrations. Post 24 h incubation with the compound the media was removed and 0.5 mg/ml MTT was added to the cells. The formazon crystals were solubilized by adding 100 μL of freshly prepared solubilization solution (20% SDS in 50% Dimethylformamide). The absorbance was measured at 570 nm using BioTeK Power Wave XS2 spectrophotometer and percent toxicity was calculated with respect to control cells with triton-X treatment.

### Antibiofilm Activity

The effect of Y5-P1 on biofilm formation of *S. aureus* ATCC33591 and ATCC 43300 was assessed using MTT assay as described previously with some modifications (Tang et al., 2011). We initially optimized the incubation time, protocol, and media conditions to study the biofilm. The cultures were grown overnight in BHI medium (supplemented with 1% sucrose) at 250 rpm in highly aerobic condition. The compound was added to a final volume of 200 μl of fresh medium in a 96-well flat-bottom polystyrene plate. Two microliters of the culture was then transferred to this broth and plate was incubated at 37°C without shaking for 30 h. The wells without any compound and the wells without any culture served as positive and negative controls, respectively. After the treatment, the planktonic bacteria were washed with PBS three times and 100 μl of fresh PBS containing MTT (0.5 mg/ml) was added. The plate was incubated for 1 h and 100 μl of the stopping solution (20% SDS in 50% DMF) was added to dissolve the formazon crystals. The plate was read in microplate reader at 570 nm. The biofilm formation in positive control wells, after subtracting the background absorbance, was considered as 100% and the antibiofilm activity of the compound at different concentrations was calculated with respect to controls wells. The experiment was conducted in four replicates. The antibiofilm potential of Y5-P1 was also predicted by a software algorithm present in *aBiofilm* database (Rajput et al., 2017), which has been designed to analyze the *in silico* antibiofilm potential of new chemical scaffolds.

## Results

### Antimicrobial Activity of Yak Milk Isolates

**Figure 1:**
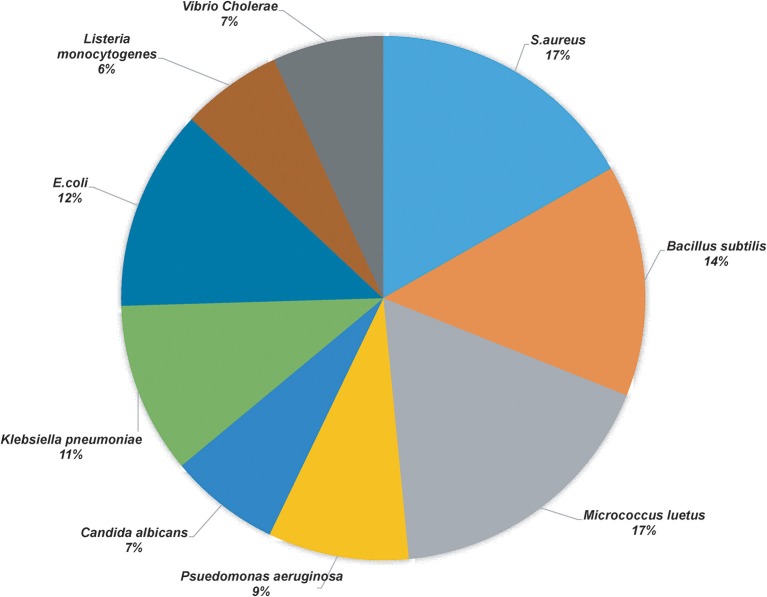
Pie chart representation of the percentage of the positive yak milk isolates in antimicrobial screening.

More than 50 isolates were screened for the antimicrobial activity against nine indicator strains. Thirty-two isolates showed positive results, of which best producers were selected based on their identification and literature if they were previously unreported for the production of antimicrobial compound(s). Table 1 shows the microbial isolates showing antimicrobial activity. Upon the analysis of the antimicrobial activities, we found that 17% of the total strains tested were active against *Staphylococcus aureus (S. aureus)* and *Micrococcus luteus*, 14% of the isolates inhibited *Bacillus subtilis*, 11 and 12% of the strains showed antimicrobial activity against *Klebsiella pneumonia* and *E. coli*, respectively, 9% of the strains were active against *Pseudomonas aeruginosa* and 7% of the strains demonstrated antimicrobial activity against *Vibrio cholera* and *Candida albicans*, 6% of the strains exhibited activity against *Listeria monocytogenes* (Figure 1).

**Table 1:** List of microbial isolates showing antimicrobial activity.

**S.No**	**Strain**	***S. aureus***	***Bacillus subtilis***	***Micrococcus luteus***	***Pseudomonas aerugenosa***	***Candida albicans***	***Klebsiella pneumoniae***	***E.coli***	***Listeria monocytogenes***	***Vibrio Cholerae***
1	Y-2	+	–	+	–	–	–	–	–	–
2	Y-5	+	+	+	–	+	+	+	+	+
3	Y-14	+	+	+	–	–	+	+	–	+
4	Y-21	+	–	+	–	–	–	+	–	+
5	Y-23	+	–	+	–	–	–	–	–	–
6	Y-25	+	+	–	–	–	–	–	–	–
7	Y-27	+	–	–	–	–	–	–	+	+
8	Y-28	+	+	+	–	–	–	+	+	+
9	Y-30	+	+	+	–	–	–	–	+	+
10	Y-31	+	–	+	–	–	+	+	–	–
11	Y-33	+	–	+	–	–	–	+	–	–
12	Y-36	–	+	–	–	–	–	–	+	–
13	Y-37	–	+	+	–	–	–	–	–	–
14	Y-38	–	–	–	–	–	–	–	–	–
15	Y-45	–	–	+	–	–	–	+	–	–
16	Y-48	–	+	+	–	–	–	–	–	+
17	Y-53	+	–	–	–	–	–	–	–	–
18	Y-51	+	+	+	+	–	+	+	–	+
19	Y-1	+	+	+	+	–	+	+	+	–
20	Y-b	+	+	–	–	–	+	+	+	+
21	LAN−4	+	+	+	+	–	+	+	+	+
22	Ya	+	+	+	+	+	+	+	–	–
23	Milk−2	+	+	+	+	+	+	+	–	–
24	An5	–	+	+	+	–	+	+	–	–
25	Y-47	+	+	+	+	–	+	+	–	–
26	Y-44	+	+	+	–	–	+	–	–	–
27	Yibs	+	+	+	+	–	+	+	–	–
28	An4	+	+	+	+	–	+	+	–	–
29	L4	+	+	+	+	–	+	+	–	–
30	Y-50	+	+	+	+	+	+	+	–	–
31	Y-52	+	–	+	–	–	+	–	–	–
32	Yes	+	+	+	+	+	+	+	–	–

^*^The antimicrobial activities of the strains (No.18–32) has been previously discussed in report (Kaur et al., 2017).

### Isolation and Characterization of the Antimicrobial Compound From Y-5 Strain

**Figure 2:**
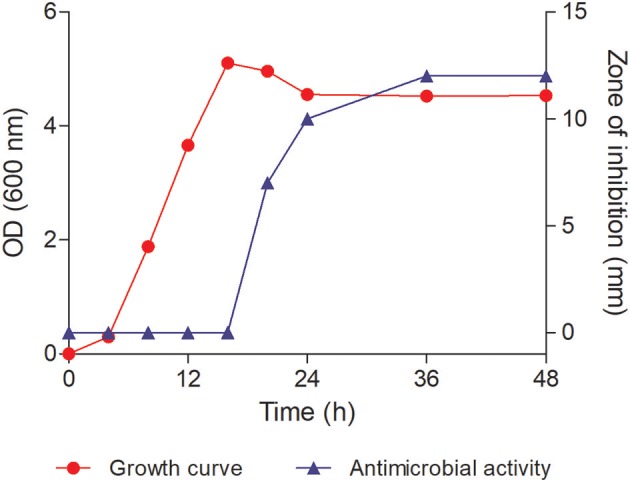
Growth curve of the strain Y-5 and its antimicrobial activity against *Bacillus subtilis* ATCC 6633. The antimicrobial activity started around 24 h of incubation.

Y-5 strain displayed inhibitory activity against both Gram-positive and Gram-negative bacteria and showed 100% similarity with *P. koreensis* (Kwon et al., 2003) based on 16S rRNA gene sequence data (GenBank accession number MK372235). Cells of the strain Y5 were Gram-negative, rod-shaped (Supplementary Figure S1). The growth curve analysis revealed that the strain Y-5 took 4 h to complete the lag phase, and reached late exponential phase in 16 h. It was observed that the strain started producing antimicrobial component(s) after 24 h of incubation (in the early stationary phase). The activity increased from mid-stationary phase to late stationary phase as shown in Figure 2. The activity was constant in late stationary phase and thus large-scale fermentation batches were harvested in this phase. The bioactivity-guided fractionation in reverse-phase HPLC of LH-active fractions revealed that one prominent peak active eluting at 10.5 min was responsible for bioactivity (Supplementary Figure S2A). This peak showed strong absorbance in the UV detector at 220 and 290 nm. The purity of the peak was more than 98% as determined by analytical HPLC (Supplementary Figure S2B). The dried compound appeared as a lemon-yellow colored powder. The purified compound showed a molecular mass of 253.0583 [M-H]^−^ in HR-ESI-MS (Supplementary Figure S2C) with negative ion mode and 255.0769 [M+H]^+^ in LC-ESI-MS (Supplementary Figure S3) with positive ion mode which led us to deduce the molecular formula as C_14_H_10_N_2_O_3_ (calculated monoisotopic mass 254.069 for [M]). This formula also satisfied the nitrogen rule of chemical compounds which lack halogen atom (even number of nitrogen for even molecular mass). FTIR analysis showed a broad intense peak at 3,429 cm^−1^ which suggested the presence of hydroxyl group of either an alcohol or a carboxylic acid (Supplementary Figure S4). The other peaks near 1,650 cm−1 confirmed the presence of a carboxylic moiety in the compound. But this information along with the molecular formula was insufficient to solve the complete structure of the compound. Therefore, one and two-dimensional NMR spectroscopy techniques were used to assign all the atoms in the molecule.

### NMR Analysis

**Figure 3:**
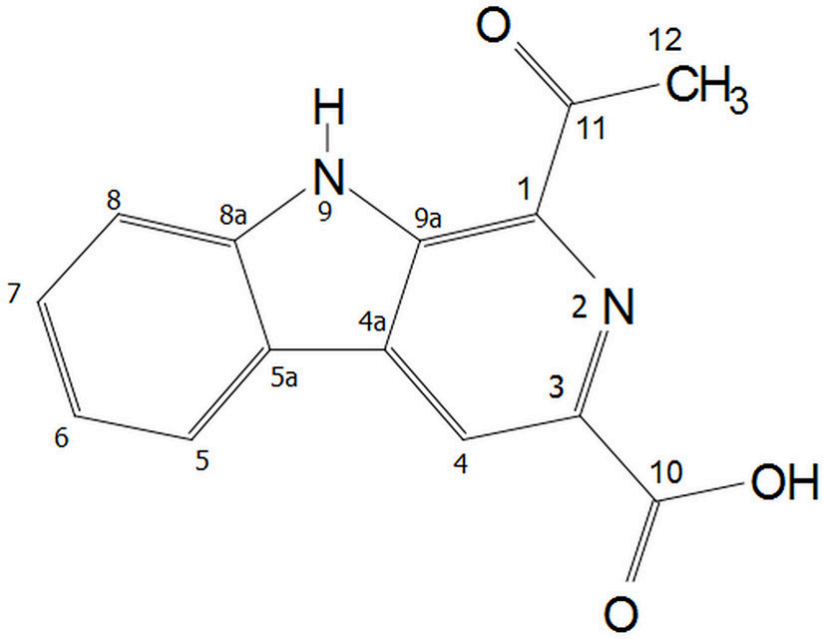
Structure of Y5-P1 (1-acetyl-9H-β-carboline-3-carboxylic acid).

In the ^1^H NMR spectrum of Y5P1, various proton signals appeared as follows: δ_H_ ppm, 12.25 (s, 1H), 9.16 (s, 1H), 8.45 (d, *J* = 7.8 Hz, 1H), 7.85 (d, *J* = 8.2 Hz, 1H), 7.64 (t, *J* = 7.58 Hz, 1H,), 7.36 (t, *J* = 7.5 Hz, 1H), 2.85 (s, 3H). Two doublets appeared at δ_H_ 8.45 (d, *J* = 7.8 Hz, 1H) and 7.85 ppm (d, *J* = 8.2 Hz, 1H) were assigned to H-5 and H-8, respectively. Two triplet signals appeared at δ_H_ 7.64 (t, *J* = 7.6 Hz, 1H, *H-7*) and 7.36 (t, *J* = 7.5 Hz, 1H, *H-6*) ppm were assigned to H-7 and H-6, respectively. A singlet observed at δ_H_ 9.16 was assigned to H-8 proton. These assignments were further confirmed using 2D ^1^H-^1^H correlation COSY spectrum. In the ^13^C NMR spectrum, various carbon signals appeared as follows: δ_C_ ppm, 201.63, 166.86, 142.22, 137.02, 135.53, 135.44, 131.94, 129.77, 122.68, 121.49, 121.40, 120.70, 113.85, and 26.25 where δ_C_ at 201.63 ppm was assigned as carbonyl of acetyl group whereas a signal at δ_C_ 166.86 ppm was due to carbonyl of carboxyl group. DEPT 135 spectrum showed that there are five CH signals, one CH_3_ signals whereas CH_2_ carbon signal is absent in the molecule, and remaining are the quaternary carbons and carbonyl carbons. The signal observed at δ_H_ 12.25 ppm was assigned as NH(9) as it showed coupling to β-carboline ring carbons in HMBC spectrum and also due to intra-molecular hydrogen bonding with carbonyl group of acetyl group assigned at C-1 of the β-carboline ring. The NMR assignments of all the atoms present in the compound are shown in Table S1. The NMR spectra are given in Supplementary Figures S5–S12. The structure of this compound characterized using mass spectroscopy, FTIR and NMR analysis is presented in Figure 3. The chemical name of the compound is 1-acetyl-9H-β-carboline-3-carboxylic acid.

### Minimum Inhibitory Concentration (MIC)

The MIC of the compound Y5-P1 was checked against different quality control strains and multidrug-resistant clinical isolates, and results are listed in Table 2. This compound was less active against Gram-positive bacteria than Gram-negative bacteria with the MIC of 62.5–125 μg/ml against all the Gram-negative bacteria tested. *B. subtilis* showed the inhibition at 125 μg/ml while *S. aureus* was inhibited at 250 μg/ml. Though MIC values were considerably higher for Gram-negative pathogens also but the MIC was consistent among most of the MDR strain tested. This data suggests that the compound may have a different target than already existing class of antibiotics.

**Table 2:** Minimum inhibitory concentration of Y5-P1 against pathogenic strains.

**Strain**	**Strain number**	**MIC (μg/ml)**
*Bacillus subtillis*	ATCC 6051	125
*S.aureus*	ATCC 25923	250
*E. coli*	ATCC 25922	62.5
*Klebsiella pneumoniae*	ATCC 700603	62.5
*Psuedomonas aeruginosa*	ATCC 27853	62.5
*Acinetobacter baumanii*	ATCC 19606	62.5
*Klebsiella pneumoniae* (MDR)	GMCH 04	62.5
*Klebsiella pneumoniae* (MDR)	GMCH 13	62.5
*Psuedomonas aeruginosa* (MDR)	GMCH 06	62.5
*Acinetobacter baumanii* (MDR)	GMCH 05	62.5
*E. coli* (MDR)	7932	62.5
*E. coli* (MDR)	9062	62.5

### Time-Kill Assay and Effect on Bacterial Morphology

**Figure 4:**
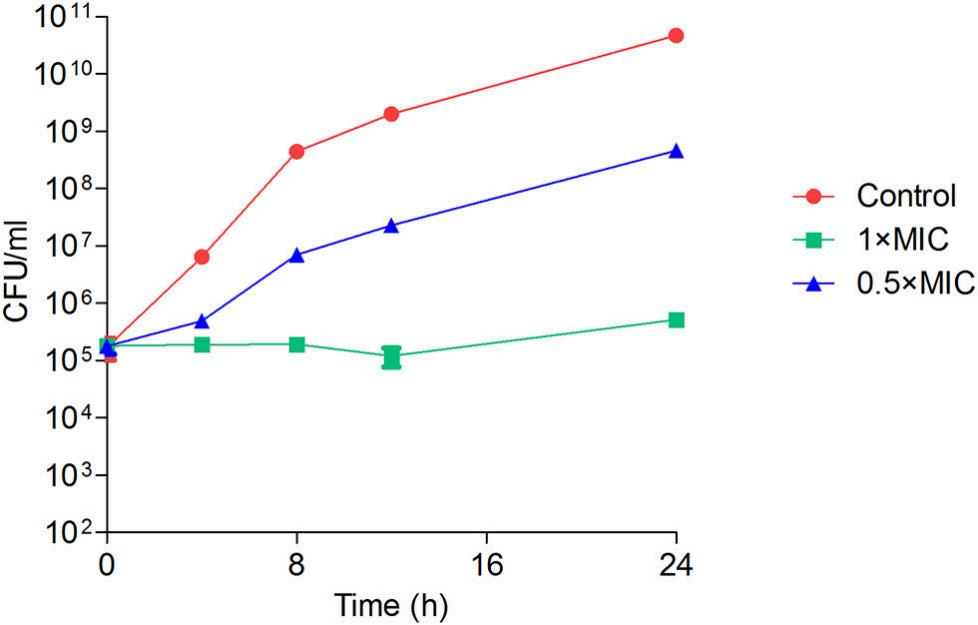
Time-kill kinetics of Y5-P1 showing the bacteriostatic action. The data are represented as mean ± SD of three replicates.

**Figure 5:**
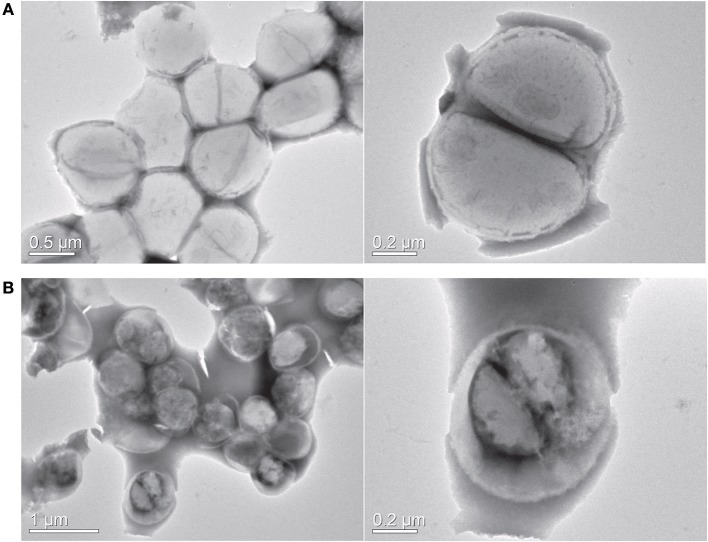
Transmission electron microscopy. **(A)** Control cells **(B)** Y5-P1 treated cells. Right panel shows the cell shape and morphology at higher magnification. Upon the treatment, the cells displayed a distorted morphology, and intracellular material is aggregated. Also, the bacteria are showing the disrupted cell division.

As visible in Figure 4, the viable number of bacteria at MIC concentration remained unchanged for 24 h, suggesting the bacteriostatic nature of this compound. At subinhibitory concentration, the bacteria multiplied, but at a slower rate when compared to untreated control. We next sought to study the effect of this compound on bacterial morphology using electron microscopy. Figure 5 shows the shape and morphology of treated and untreated bacteria. The compound caused aggregation of intracellular material in the cell, visible as dark irregular matter. Also the cell shape was distorted after the treatment. The effect on membrane or cell wall of the bacteria was not visible in microscopy, but the compound treatment altered the overall morphology of the cells. The cell division was also disturbed after treatment; cleavage furrow was not clearly visible in the treated bacteria.

### Mammalian Cytotoxicity

**Figure 6:**
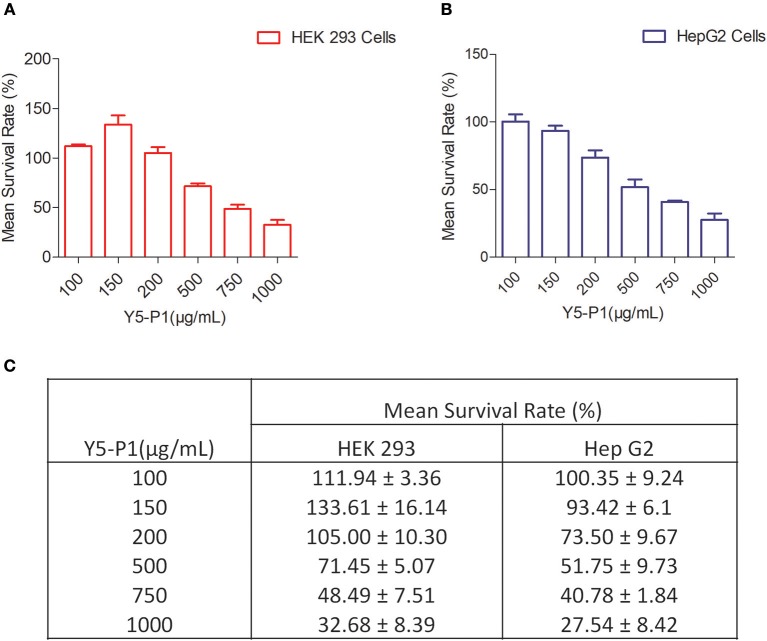
Mammalian Toxicity of Y5-P1 compound in **(A)** HEK 293 cells; **(B)** Hep G2 cells **(C)** The toxicity results are represented in tabular form.

To evaluate the mammalian toxicity of this compound, we performed MTT assay in HEK293T and HEPG2 cell lines with different concentrations. Results indicated that the IC_50_ of compound Y5-P1 was ~10-folds higher than its MIC value. The IC_50_ value was ~ 750 and ~ 500 μg/ml for HEK-293T cell line and HEPG2 cell line, respectively as shown in Figure 6.

### Antibiofilm Activity

**Figure 7:**
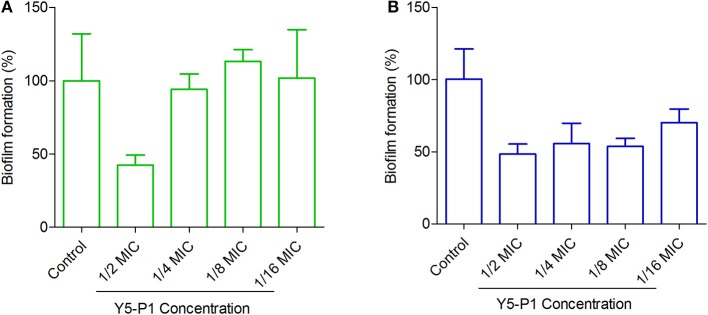
Antibiofilm activity of Y5-P1 in MRSA strains. **(A)** *S. aureus* ATCC 33591 and **(B)** *S. aureus* ATCC 43300. The compound inhibited ~50% of the biofilm at subtoxic concentrations. The experiment was performed in four replicates and presented as Mean ± SD.

Although the compound Y5-P1 had moderate activity in *S. aureus* strains, it exhibited antibiofilm properties in MRSA strains at sublethal concentrations (Figure 7). In MRSA strain ATCC 33591, the compound inhibited almost 50% of the biofilm at half of its MIC concentration and below this concentration, no antibiofilm effect was observed. In contrast, the compound inhibited the biofilm formation in ATCC 43300 strain by 50% even at one-eighth of its MIC. At 1/16th of MIC concentration, the inhibition was around 45%. Such effects of organic compounds, which are bacteriostatic in nature, on bacterial biofilm have been recently reported for cytochalasins family of compounds (Yuyama et al., 2018). Also, the mechanism of action and target for antibiofilm activity of this compound is not known and could provide further insights into designing of better synthetic analogs of this scaffold. This part is beyond the scope of the present study.

## Discussion

This study aimed at the discovery of unexplored antimicrobial compounds from an untapped or unique niche i.e., yak milk. We screened fifty bacterial isolates for their ability to inhibit the growth of test strains and sixty-five percent of the cultures successfully inhibited at least one of the indicator strains. More than 50% of the isolates belonged to lactic acid bacteria which are known to produce antimicrobial peptides or other bioactive secondary metabolites (Sawa et al., 2013; Perez et al., 2014; Mirkovic et al., 2016). We have also shown in our previous paper (Kaur et al., 2017) the antimicrobial activity of seventeen lactic acid bacteria out of fifty isolates. Out of thirty-two positive isolates having antimicrobial activity, most of them had been reported in literature to possess inhibitory activity, whereas some of the isolates exhibited inconsistent activity. In the present study, we selected the strain Y5 for the purification of the antimicrobial compound due to its consistent activity and because of the fact that this strain was unreported for any antimicrobial activity. Y5 was identified as *P. koreensis* and was active against both Gram-positive and Gram-negative bacteria. This genus is well-reported to synthesize a diverse array of antimicrobial compounds such as phenazine-1-carboxylic acid, pyoluteorin, mupirocin, promysalin, phenazine-1-carboxamide, viscosin amide, 2,4-Diacetylphloroglucinol, Pyocyanin, Pyrrolnitrin, and tesin (Howell and Stipanovic, 1980; Shanahan et al., 1992; Pierson and Pierson, 1996; Chin-a-Woeng et al., 2003; Hu et al., 2005; Huang et al., 2009; Li et al., 2011; Gao et al., 2014). At lower temperatures, *Pseudomonas* spp. are predominant in the raw milk as reported by several researchers (Capodifoglio et al., 2016; Meng et al., 2017, 2018). These bacteria may transfer to milk from the surrounding but they are natural inhabitant of raw milk at low storage conditions. They are also responsible for the spoilage of the milk in some cases. At lower temperatures, *Pseudomonas* can outgrow the other bacteria by more than 50% of total population. The yak milk diversity was not screened previously for antimicrobial activity. Our main objective was to isolate the bacteria from unusual niche or untapped source and check their antimicrobial potential.We successfully isolated and characterized one β-carboline derivative (Y5-P1) from Y5 strain. The compound Y5-P1 appeared as yellowish in color and had a molecular mass of 254 Da. With the aid of HRMS, FTIR and NMR spectroscopy, it was identified as1-acetyl-9H-β-carboline-3-carboxylic acid. This compound contains an indole ring which is present in many antibiotics and is responsible for antimicrobial activity (Panchal et al., 2009; Williams et al., 2013; Lepri et al., 2016). Another derivative of this class, i.e., 1-Acetyl-9H-β-carboline was reported from marine actinomycete *Streptomyces* sp. (Shin et al., 2010). This compound showed similar activity as reported in the present study. Also, the compound acted in synergy with β-Lactams against methicillin-resistant *S. aureus* (MRSA). They had reported the first time a β-carboline synthesized by microorganisms. β-carboline compounds belong to the family indole alkaloids which are pharmacologically active natural ingredients produced by many plant species (Cao et al., 2007). They are reported as bioactive molecules having bioactivities, such as cytotoxic, antiviral, antimicrobial, anti-inflammatory, antiserotonin, and enzyme inhibition (Cao et al., 2007; Milen and Ábrányi-Balogh, 2016). Y5-P1 showed the MIC in the range of 62.5–250 μg/ml against multidrug-resistant bacteria. Time-kill assay revealed that Y5-P1 is bacteriostatic in nature and caused alteration in bacterial shape and morphology upon treatment. One example of the potent antimicrobial agent having bacteriostatic action is tetracycline (Chopra and Roberts, 2001). Thus, Y5-P1 represents a scaffold which could be synthetically derivatized to improve the potency. The compound had reduced mammalian cytotoxicity in two cell lines (500 and 750 μg/ml against HEK293T and HepG2 cell lines, respectively). This derivative i.e., 1-acetyl-9H-β-carboline-3-carboxylic acid is different from previously reported 1-Acetyl-9H-β-carboline due to the presence of carboxylic moiety and was first reported from a plant *Vestia lycioides*, family Solanaceae in 1980 (Faini et al., 1980). We, in this study, report the synthesis of this derivative by a microorganism that is an inhabitant of an unusual niche. The β-carboline scaffold could be an important structure in designing new compounds (Trujillo et al., 2007) which would be very much effective and less toxic to humans. One example of a marketed drug based on indole nucleus is tadalafil.

Additionally, the compound showed antibiofilm activity in MRSA strains at subtoxic concentrations. We also predicted the antibiofilm propensity of Y5-P1 using *aBiofilm* predictor program and found that this compound had high probability of having antibiofilm potential (Supplementary Figure S13). Moreover, no similar compound was found in the database. These data suggest that Y5-P1 represents a new chemical scaffold having antibiofilm properties that should be further optimized through medicinal chemistry approaches to improve the antibiofilm activity. The target of this compound is not known in bacteria. Further investigations in the target identification may lead to a novel target for drug development. This study highlights the importance of natural products as a source of antimicrobial compounds. Microbes residing in uncommon environments or untapped sources possess a vast repertoire of the antimicrobial substances which may represent a promising drug candidate or new scaffold for drug designing against highly resistant pathogens.

## Accession Numbers

16S rRNA gene sequence was submitted to GenBank/EMBL/DDBJ under accession number MK372235. The strain Y5 was deposited in Microbial Type Culture Collection (MTCC) repository, India and MTCC No. 12911 was obtained.

## Ethics Statement

This article does not contain any studies with human participants or animals performed by any of the authors.

## Author Contributions

MK, MJ, HS, and RT performed the experiments, data analysis. MK and MJ wrote and edited the manuscript. SJ did structural characterization. CS and NS performed and analyzed toxicity studies. AP, HN, and SM designed and supervised the study. All authors approved the manuscript before submission.

### Conflict of Interest Statement

The authors declare that the research was conducted in the absence of any commercial or financial relationships that could be construed as a potential conflict of interest.

## References

[B1] An D. Dong X. Dong Z. (2005). Prokaryote diversity in the rumen of yak (*Bos grunniens*) and Jinnan cattle (*Bos taurus*) estimated by 16S rDNA homology analyses. Anaerobe 11, 207–215. 10.1016/j.anaerobe.2005.02.001 16701570

[B2] Andersson D. I. Hughes D. (2010). Antibiotic resistance and its cost: is it possible to reverse resistance? Nat. Rev. Microbiol. 8, 260–271. 10.1038/nrmicro2319 20208551

[B3] Bao Q. Yu J. Liu W. Qing M. Wang W. Chen X. (2012). Predominant lactic acid bacteria in traditional fermented yak milk products in the Sichuan Province of China. Dairy Sci. Technol. 92, 309–319. 10.1007/s13594-012-0061-x

[B4] Bowen W. (2017). Harnessing Microbial Machinery in the Hunt for New Antibiotics. Cambridge. Available online at: https://www.ttp.com/blog/harnessing-microbial-machinery-in-the-hunt-for-new-antibiotics

[B5] Bush K. Courvalin P. Dantas G. Davies J. Eisenstein B. Huovinen P.. (2011). Tackling antibiotic resistance. Nat. Rev. Microbiol. 9, 894–896. 10.1038/nrmicro2693 22048738 PMC4206945

[B6] Cao R. Peng W. Wang Z. Xu A. (2007). β-Carboline alkaloids: biochemical and pharmacological functions. Curr. Med. Chem. 14, 479–500. 10.2174/092986707779940998 17305548

[B7] Capodifoglio E. Vidal A. M. C. Lima J. S. Bortoletto F. D'abreu L. F. Netto A.S. (2016). Lipolytic and proteolytic activity of Pseudomonas spp. isolated during milking and storage of refrigerated raw milk. J. Dairy Sci. 99, 5214–5223. 10.3168/jds.2015-10453 27085402

[B8] Centers for Disease Control and Prevention (2014). Antibiotic Resistance Threats in the United States, 2013. Atlanta: CDC.

[B9] Challinor V. L. Bode H. B. (2015). Bioactive natural products from novel microbial sources. Ann. N. Y. Acad. Sci. 1354, 82–97. 10.1111/nyas.12954 26509922

[B10] Chellat M. F. Riedl R. (2017). Pseudouridimycin: the first nucleoside analogue that selectively inhibits bacterial RNA polymerase. Angew. Chem. Int. Edn. 56, 13184–13186. 10.1002/anie.201708133 28895263

[B11] Chin-a-Woeng T. F. Bloemberg G. V. Lugtenberg B. J. (2003). Phenazines and their role in biocontrol by Pseudomonas bacteria. N. Phytol. 157, 503–523. 10.1046/j.1469-8137.2003.00686.x 33873412

[B12] Chopra I. Roberts M. (2001). Tetracycline antibiotics: mode of action, applications, molecular biology, and epidemiology of bacterial resistance. Microbiol. Mol. Biol. Rev. 65, 232–260. 10.1128/MMBR.65.2.232-260.2001 11381101 PMC99026

[B13] Clardy J. Fischbach M. A. Walsh C. T. (2006). New antibiotics from bacterial natural products. Nat. Biotechnol. 24, 1541–1550. 10.1038/nbt1266 17160060

[B14] Faini F. Torres R. Delle Monache F. Marini-Bettolo G. Castillo M. (1980). 1-Acetyl-3-carboxy-β-carboline, a new acid and other constituents of Vestia lycioides. Planta Med. 38, 128–132. 10.1055/s-2008-1074847

[B15] Gao S. S. Hothersall J. Wu J. E. Murphy A. C. Song Z. Stephens E. R.. (2014). Biosynthesis of mupirocin by Pseudomonas fluorescens NCIMB 10586 involves parallel pathways. J. Am. Chem. Soc. 136, 5501–5507. 10.1021/ja501731p 24625190

[B16] Gelband H. Molly Miller P. Pant S. Gandra S. Levinson J. Barter D. (2015). State of the World's Antibiotics, 2015. Washington, DC: CDDEP Available online at: https://cddep.org/wp-content/uploads/2017/06/swa_edits_9.16.pdf

[B17] Giorgi E. E. (2016). The antibacterial resistance threat. Are We Heading Toward a Post-Antibiotic Era? in Los Alamos National Lab. (LANL) (Los Alamos: NM). 10.2172/1245551

[B18] Hover B. M. Kim S.-H. Katz M. Charlop-Powers Z. Owen J. G. Ternei M. A.. (2018). Culture-independent discovery of the malacidins as calcium-dependent antibiotics with activity against multidrug-resistant Gram-positive pathogens. Nat. Microbiol. 3, 415–422. 10.1038/s41564-018-0110-1 29434326 PMC5874163

[B19] Howell C. Stipanovic R. (1980). Suppression of *Pythium ultimum*-induced damping-off of cotton seedlings by *Pseudomonas fluorescens* and its antibiotic, pyoluteorin. Phytopathology 70, 712–715. 10.1094/Phyto-70-712

[B20] Hu H.-B. Xu Y.-Q. Feng C. Xue H. Z. Hur B.-K. (2005). Isolation and characterization of a new fluorescent Pseudomonas strain that produces both phenazine 1-carboxylic acid and pyoluteorin. J. Microbiol. Biotechnol. 15, 86–90.

[B21] Huang J. Xu Y. Zhang H. Li Y. Huang X. Ren B.. (2009). Temperature-dependent expression of phzM and its regulatory genes lasI and ptsP in rhizosphere isolate Pseudomonas sp. strain M18. Appl. Environ. Microbiol. 75, 6568–6580. 10.1128/AEM.01148-09 19717631 PMC2765144

[B22] Kaur M. Singh H. Jangra M. Kaur L. Jaswal P. Dureja C.. (2017). Lactic acid bacteria isolated from yak milk show probiotic potential. Appl. Microbiol. Biotechnol. 101, 7635–7652. 10.1007/s00253-017-8473-4 28879447

[B23] Kwon S. W. Kim J. S. Park I. C. Yoon S. H. Park D. H. Lim C. K.. (2003). *Pseudomonas koreensis* sp. nov., *Pseudomonas umsongensis sp*. nov. and *Pseudomonas jinjuensis* sp. nov., novel species from farm soils in Korea. Int. J. Syst. Evolutionary Microbiol. 53, 21–27. 10.1099/ijs.0.02326-0 12656147

[B24] Lepri S. Buonerba F. Goracci L. Velilla I. Ruzziconi R. Schindler B. D.. (2016). Indole based weapons to fight antibiotic resistance: a structure–activity relationship study. J. Med. Chem. 59, 867–891. 10.1021/acs.jmedchem.5b01219 26757340

[B25] Li W. Estrada-De Los Santos P. Matthijs S. Xie G.-L. Busson R. Cornelis P.. (2011). Promysalin, a salicylate-containing *Pseudomonas putida* antibiotic, promotes surface colonization and selectively targets other Pseudomonas. Chem. Biol. 18, 1320–1330. 10.1016/j.chembiol.2011.08.006 22035801

[B26] Ling L. L. Schneider T. Peoples A. J. Spoering A. L. Engels I. Conlon B. P.. (2015). A new antibiotic kills pathogens without detectable resistance. Nature 517, 455–459. 10.1038/nature14098 25561178 PMC7414797

[B27] Liu H. Ren F. Jiang L. Ma Z. Qiao H. Zeng S. (2011). Short communication: fatty acid profile of yak milk from the Qinghai-Tibetan plateau in different seasons and for different parities. J. Dairy Sci. 94, 1724–1731. 10.3168/jds.2010-3749 21426960

[B28] Luming D. Ruijun L. Zhanhuan S. Changting W. Yuhai Y. Songhe X. (2008). Feeding behaviour of yaks on spring, transitional, summer and winter pasture in the alpine region of the Qinghai–Tibetan plateau. Appl. Anim. Behav. Sci. 111, 373–390. 10.1016/j.applanim.2007.06.008

[B29] Meng L. Liu H. Dong L. Zheng N. Xing M. Zhang Y.. (2018). Identification and proteolytic activity quantification of Pseudomonas spp. isolated from different raw milks at storage temperatures. J. Dairy Sci. 101, 2897–2905. 10.3168/jds.2017-13617 29398021

[B30] Meng L. Zhang Y. Liu H. Zhao S. Wang J. Zheng N. (2017). Characterization of *Pseudomonas* spp. and associated proteolytic properties in raw milk stored at low temperatures. Front. Microbiol. 8:2158. 10.3389/fmicb.2017.02158 29167660 PMC5682325

[B31] Milen M. Ábrányi-Balogh P. (2016). Synthesis of β-carbolines (microreview). Chem. Heterocyclic Compounds 52, 996–998. 10.1007/s10593-017-1997-9

[B32] Mirkovic N. Polovic N. Vukotic G. Jovcic B. Miljkovic M. Radulovic Z. (2016). Lactococcus lactis LMG2081 produces two bacteriocins: a non-lantibiotic and a novel lantibiotic. Appl. Environ. Microbiol. 82, 2555–2562. 10.1128/AEM.03988-15 26896142 PMC4959506

[B33] O'neill J. (2016). The Review on Antimicrobial Resistance. 2016. Antimicrobial resistance: tackling a crisis for the health and wealth of nations. HM Government, London, United Kingdom.

[B34] Panchal R. G. Ulrich R. L. Lane D. Butler M. M. Houseweart C. Opperman T.. (2009). Novel broad-spectrum bis-(imidazolinylindole) derivatives with potent antibacterial activities against antibiotic-resistant strains. Antimicrob. Agents Chemother. 53, 4283–4291. 10.1128/AAC.01709-08 19635954 PMC2764145

[B35] Perez R. H. Zendo T. Sonomoto K. (2014). Novel bacteriocins from lactic acid bacteria (LAB): various structures and applications, in Microbial cell factories: BioMed Central (Egmond aan Zee), S3 10.1186/1475-2859-13-S1-S3 PMC4155820 25186038

[B36] Pierson L. S. Pierson E. A. (1996). Phenazine antibiotic production in *Pseudomonas aureofaciens*: role in rhizosphere ecology and pathogen suppression. FEMS Microbiol. Lett. 136, 101–108. 10.1111/j.1574-6968.1996.tb08034.x

[B37] Rajput A. Thakur A. Sharma S. Kumar M. (2017). aBiofilm: a resource of anti-biofilm agents and their potential implications in targeting antibiotic drug resistance. Nucl. Acids Res. 46, D894–D900. 10.1093/nar/gkx1157 29156005 PMC5753393

[B38] Sangma S. (2010). Optimization of the level of guar gum in low fat yak milk paneer. J. Stored Product. Postharvest Res. 1, 9–12.

[B39] Sawa N. Koga S. Okamura K. Ishibashi N. Zendo T. Sonomoto K. (2013). Identification and characterization of novel multiple bacteriocins produced by L actobacillus sakei D98. J. Appl. Microbiol. 115, 61–69. 10.1111/jam.12226 23594273

[B40] Shanahan P. O'sullivan D. J. Simpson P. Glennon J. D. O'gara F. (1992). Isolation of 2, 4-diacetylphloroglucinol from a fluorescent pseudomonad and investigation of physiological parameters influencing its production. Appl. Environ. Microbiol. 58, 353–358. 16348633 10.1128/aem.58.1.353-358.1992 PMC195214

[B41] Shin H. J. Lee H.-S. Lee D.-S. (2010). The synergistic antibacterial activity of 1-acetyl-beta-carboline and beta-lactams against methicillin-resistant *Staphylococcus aureus* (MRSA). J. Microbiol. Biotechnol. 20, 501–505. 20372018

[B42] Tang H.-J. Chen C.-C. Ko W.-C. Yu W.-L. Chiang S.-R. Chuang Y.-C. (2011). *In vitro* efficacy of antimicrobial agents against high-inoculum or biofilm-embedded meticillin-resistant *Staphylococcus aureus* with vancomycin minimal inhibitory concentrations equal to 2 μg/mL (VA2-MRSA). Int. J. Antimicrob. Agents 38, 46–51. 10.1016/j.ijantimicag.2011.02.013 21549575

[B43] Thaker M. N. Wang W. Spanogiannopoulos P. Waglechner N. King A. M. Medina R.. (2013). Identifying producers of antibacterial compounds by screening for antibiotic resistance. Nat. Biotechnol. 31, 922–927. 10.1038/nbt.2685 24056948

[B44] Trujillo J. I. Meyers M. J. Anderson D. R. Hegde S. Mahoney M. W. Vernier W. F.. (2007). Novel tetrahydro-β-carboline-1-carboxylic acids as inhibitors of mitogen activated protein kinase-activated protein kinase 2 (MK-2). Bioorganic Med. Chem. Lett. 17, 4657–4663. 10.1016/j.bmcl.2007.05.070 17570666

[B45] Williams J. D. Nguyen S. T. Gu S. Ding X. Butler M. M. Tashjian T. F.. (2013). Potent and broad-spectrum antibacterial activity of indole-based bisamidine antibiotics: synthesis and SAR of novel analogs of MBX 1066 and MBX 1090. Bioorganic Med. Chem. 21, 7790–7806. 10.1016/j.bmc.2013.10.014 24239389 PMC3906850

[B46] Yang L. Chen J. Cheng X. Xi D. Yang S. Deng W.. (2010). Phylogenetic analysis of 16S rRNA gene sequences reveals rumen bacterial diversity in Yaks (Bos grunniens). Molecular Biol. Rep. 37, 553–562. 10.1007/s11033-009-9794-x 19757178

[B47] Yuyama K. Wendt L. Surup F. Kretz R. Chepkirui C. Wittstein K.. (2018). Cytochalasans act as inhibitors of biofilm formation of *Staphylococcus aureus*. Biomolecules 8:129. 10.3390/biom8040129 30380779 PMC6316226

[B48] Zipperer A. Konnerth M. C. Laux C. Berscheid A. Janek D. Weidenmaier C.. (2016). Human commensals producing a novel antibiotic impair pathogen colonization. Nature 535, 511–516. 10.1038/nature18634 27466123

