# Supplementary material for: Pseudomonas koreensis Recovered From Raw Yak Milk Synthesizes a β-Carboline Derivative With Antimicrobial Properties
Source: Front Microbiol. 2019 Jul 29;10:1728. doi: 10.3389/fmicb.2019.01728 (PMC6681700; doi:10.3389/fmicb.2019.01728)
Supplement: Supplementary file 1 [file Data_Sheet_1.PDF]

Supplemantary information

Pseudomonas koreensis recovered from raw yak milk synthesizes a β-carboline derivative with antimicrobial properties

Manpreet Kaur[1,2,3,4], Manoj Jangra[1], Harjodh Singh[2,3,4], Rushikesh Tambat[1], Nittu Singh[5], Sanjay M Jachak[6], Sunita Mishra[3,4], Charu Sharma[5], Hemraj Nandanwar[1,4*], Anil Kumar Pinnaka[2,4*]

[1]Clinical Microbiology & Bioactive Screening Laboratory, Council of Scientific & Industrial Research -Institute of Microbial Technology, Sector -39A, Chandigarh, India

[2]MTCC-Microbial Type Culture Collection & Gene Bank, CSIR-Institute of Microbial Technology, Chandigarh-160036, India

[3]CSIR-Central Scientific Instruments Organisation, Sector 30-C, Chandigarh-160030, India

[4]Academy of Scientific and Innovative Research, (AcSIR), CSIR- New Delhi, India

[5]CSIR- Institute of Microbial Technology, Chandigarh-160036, India

[6]Department of Natural Products, National Institute of Pharmaceutical Education and Research (NIPER) Sector-67, SAS Nagar (Mohali), India

Address for correspondence

[*]Dr. P. Anil Kumar
MTCC-Microbial Type Culture Collection & Gene Bank, CSIR-Institute of Microbial Technology, Chandigarh-160036, India
E-mail: apinnaka@imtech.res.in
Telephone: +91-172-6665728

[*]Co-Correspondence

Dr. Hemraj Nandanwar
Clinical Microbiology and Bioactive Screening Laboratory, CSIR - Institute of Microbial Technology, Sector -39A, Chandigarh, India, 160036
E-mail: hemraj@imtech.res.in
Telephone: +91-172-6665338 Fax: +91-172-2690585/2690632

Supplementary Tables

Table S1 ^{1}H (400 MHz) and ^{13}C (100 MHz) NMR Data (δ in ppm) of Compound Y5P1

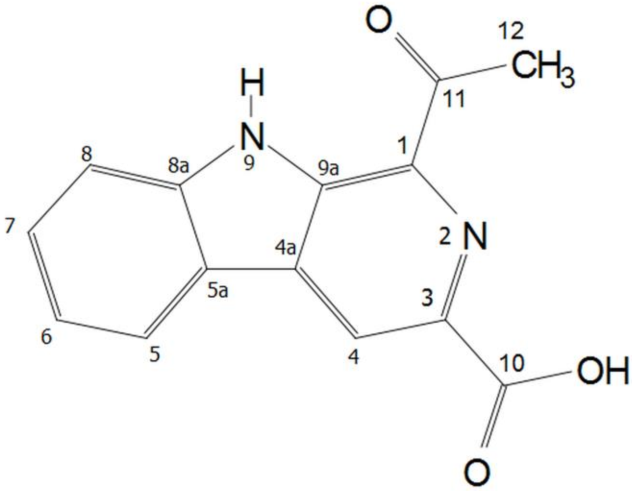

No.	δ_C, type	δ_H, mult. (*J* in Hz)	COSY	HMBC
1	135.53, C			
3	137.02, C			
4	121.49, CH	9.16 (s)		C-5a, -4a, -9a, -3
4a	131.94, C			
5	122.68, CH	8.45 (d, *J* = 7.8 Hz,)	H-6	C-5a, -7, -4a, 8a
5a	120.70, C			
6	121.40, CH	7.35 (t, *J* = 7.5 Hz)	H-5, -7	C-8, -5a
7	129.77, CH	7.64 (t, *J* = 7.6 Hz)	H-6, -8	C-8, -5, -8a
8	113.85, CH	7.85 (d, *J* = 8.2 Hz)	H-7	C-5a, -6
8a	142.73, C			
9		12.25 (s, 1H, N*H*)		C-8a, -5a, 4a, -9a
9a	135.44, C			
10	166.86, C=O			
11	201.63, C=O			
12	26.25, CH_3	2.85 (s, 3H, COCH_3)		C-1, -11

Supplementary Figures

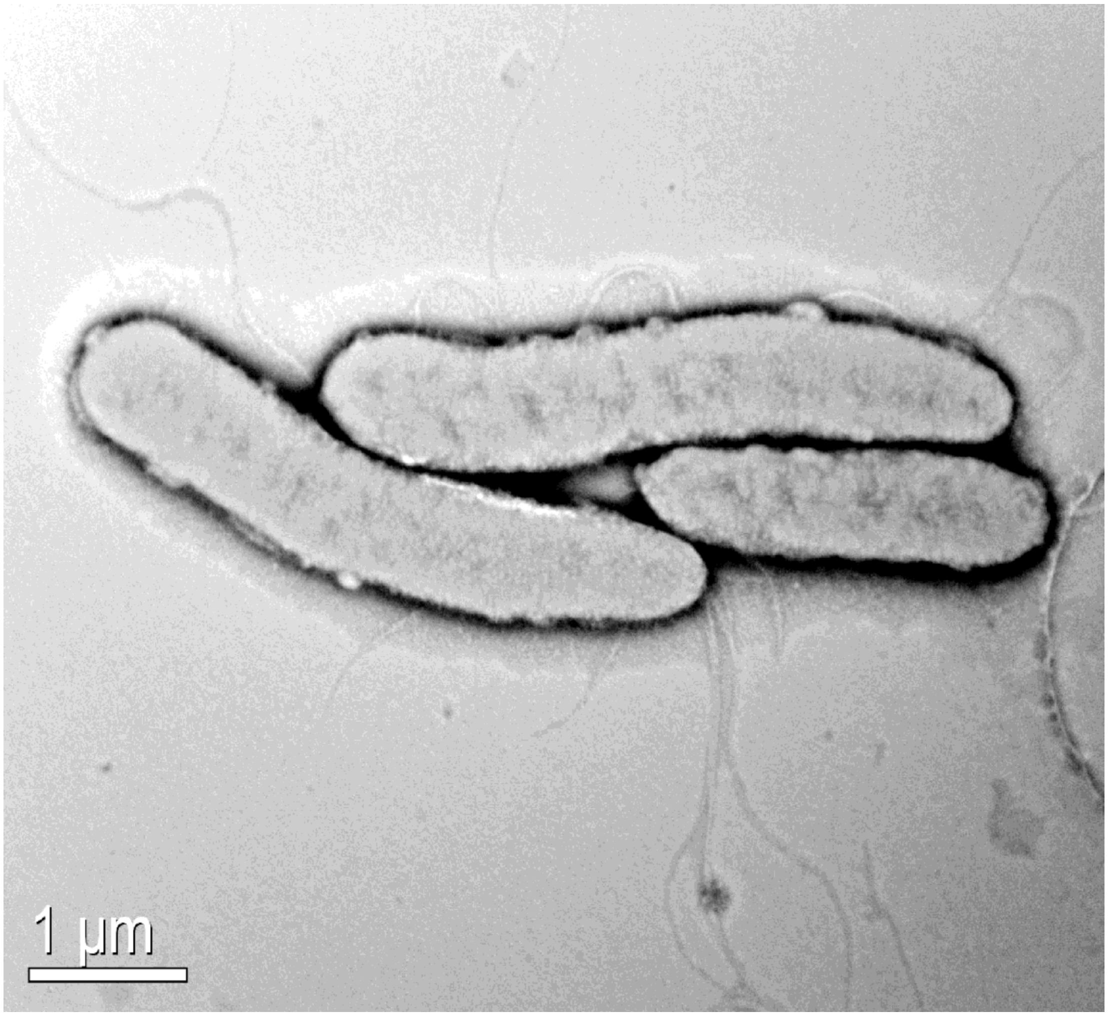

Supplementary Figure S1 Electron micrograph of cells of strain Y5, Bar 1.0μm

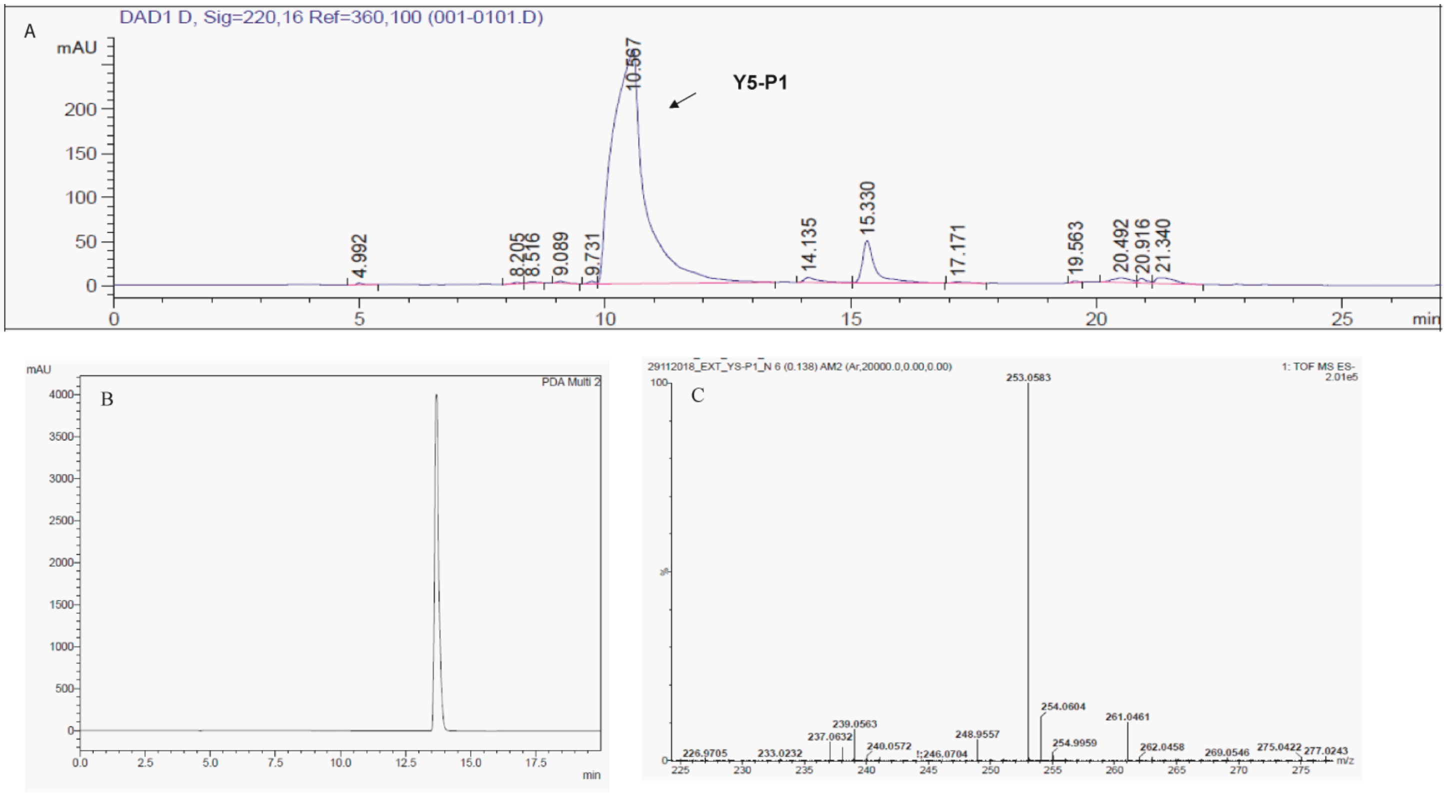

Supplementary Figure S2 (A) Reversed-phase HPLC chromatogram of LH active fraction showing the peak of Y5-P1; (B) HPLC profile of the purified compound Y5 -P1; (C) HR-ESI-MS profile of the purified peak of antimicrobial compound of Y5-P1

User Chromatograms

Fragmentor Voltage 150 **Collision Energy** 0 **Ionization Mode** ESI

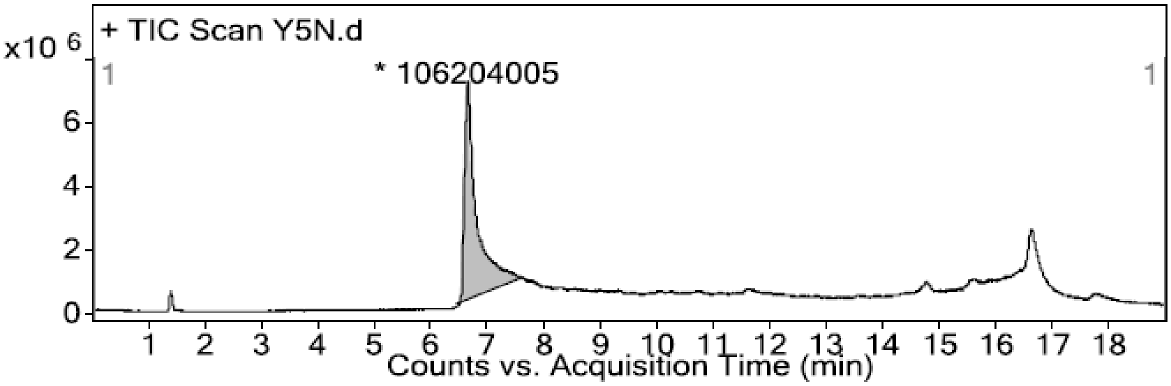

Integration Peak List

Start	RT	End	Height	Area	Area %
6.45	6.63	7.64	6879229	106204005	100

User Spectra

Spectrum Source	Fragmentor Voltage	Collision Energy	Ionization Mode
Peak (1) in "+ TIC Scan"	150	0	ESI

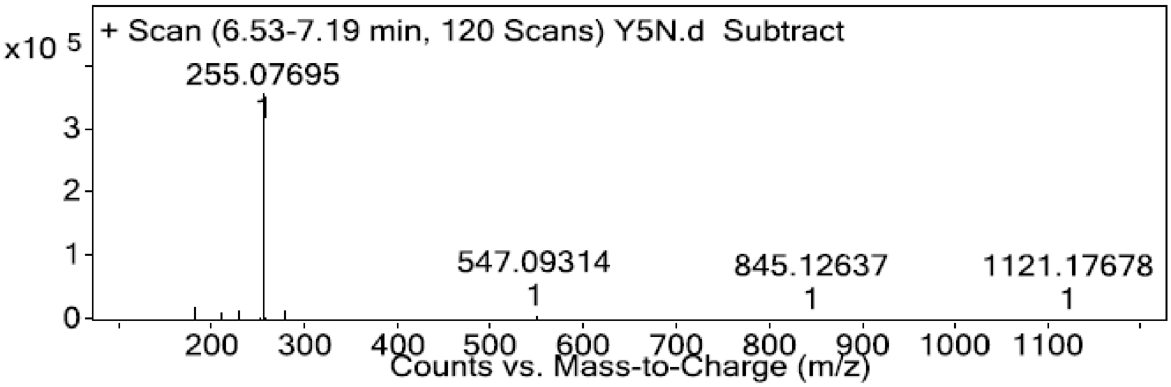

Supplementary Figure S3 LC chromatogram and MS spectrum of Y5-P1 in ESI mode showing the $[M+H]^+$ mass of 255.07695 Da.

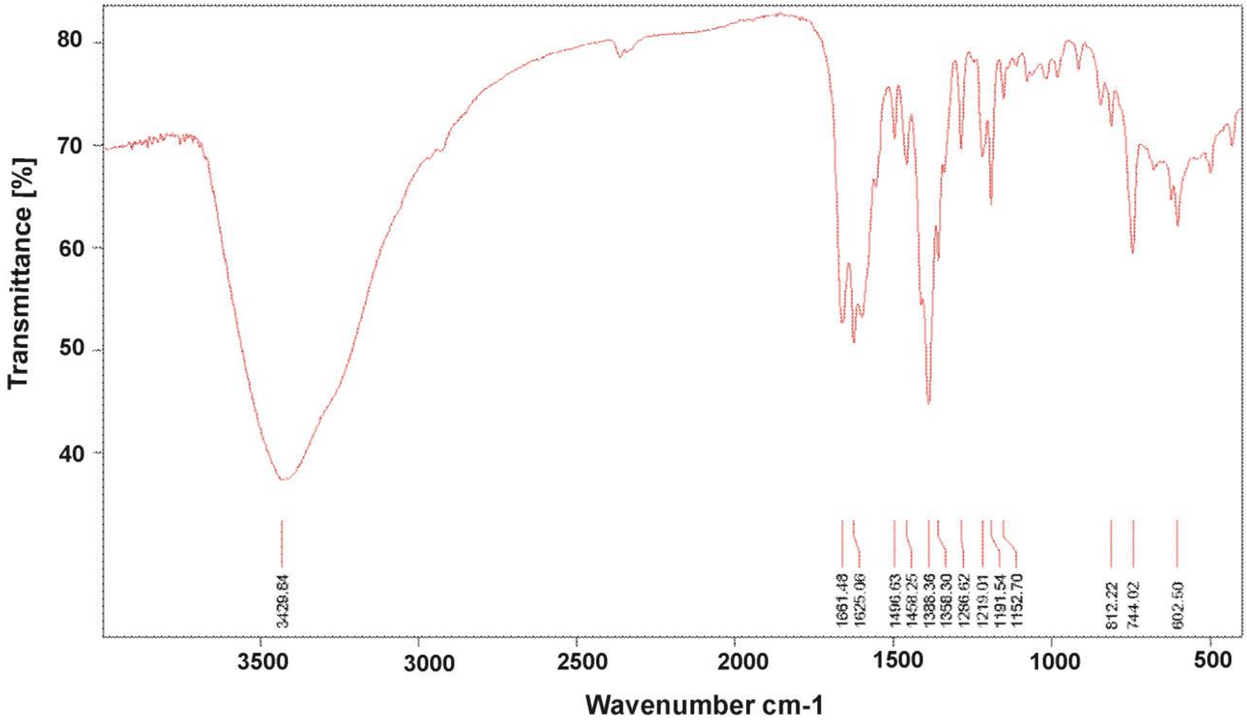

Supplementary Figure S4 FTIR spectra of pure compound Y5-P1

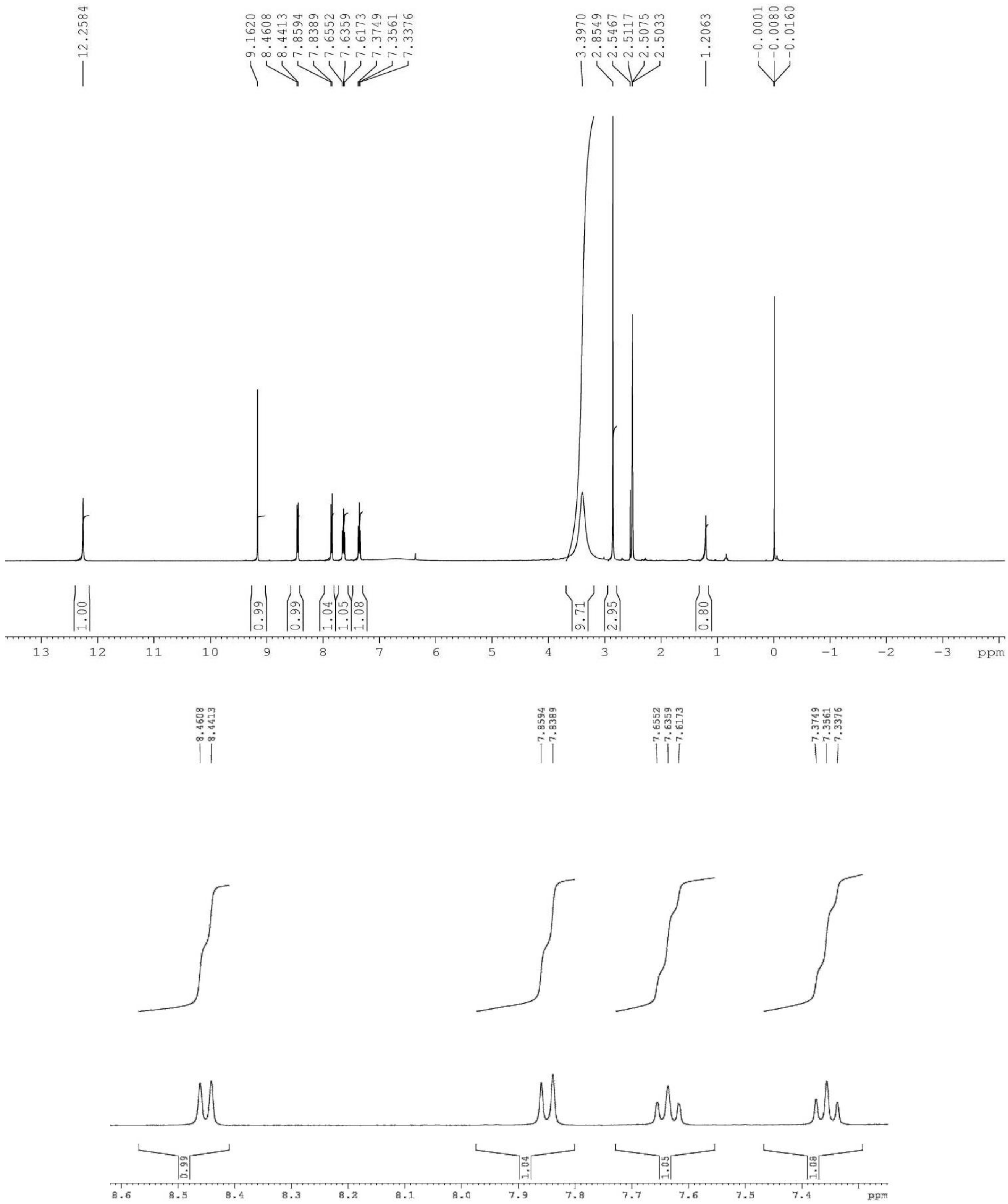

Supplementary Figure S5 ^{1}H NMR (Y5-P1)

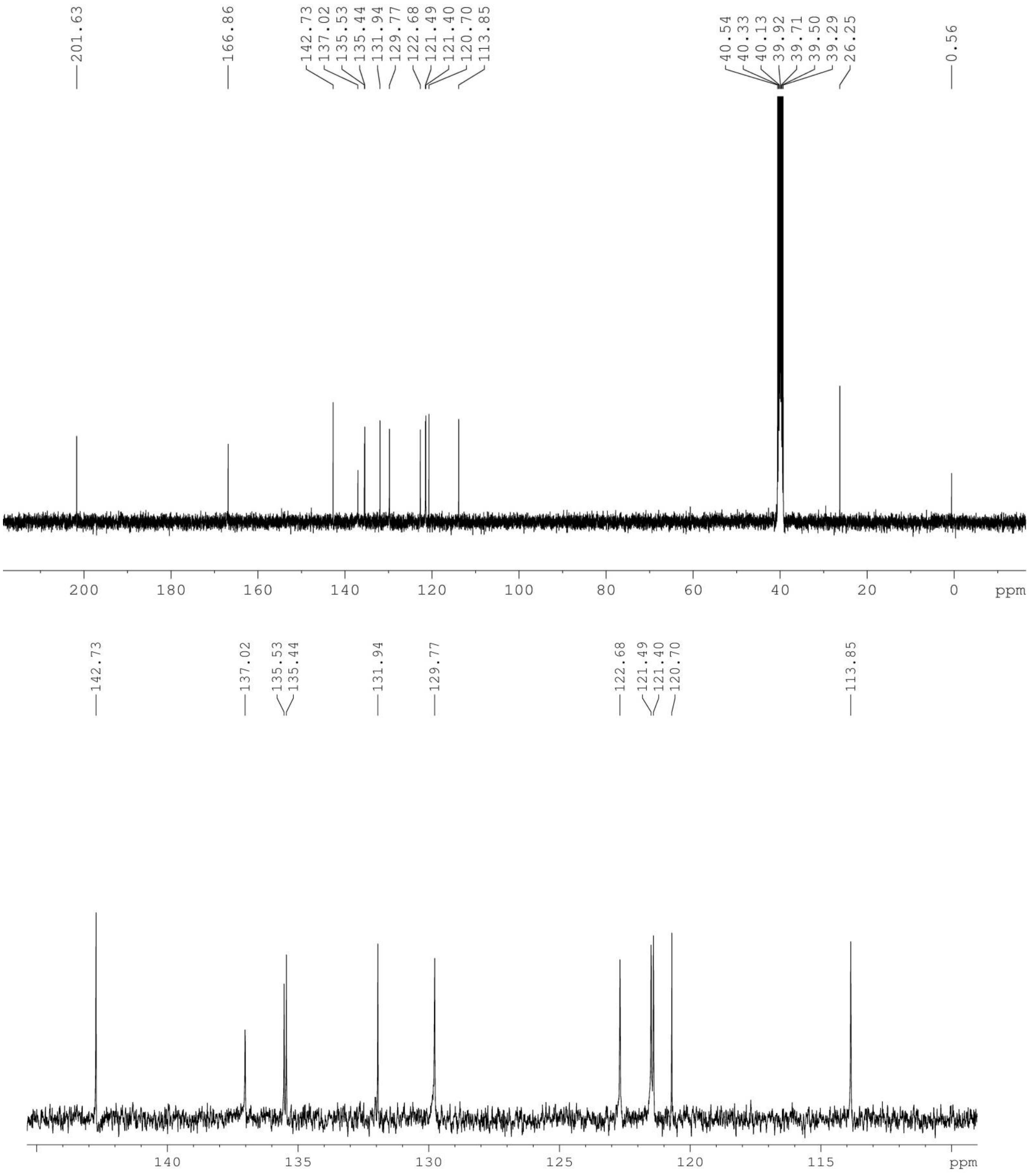

Supplementary Figure S6 ¹³C NMR (Y5-P1)

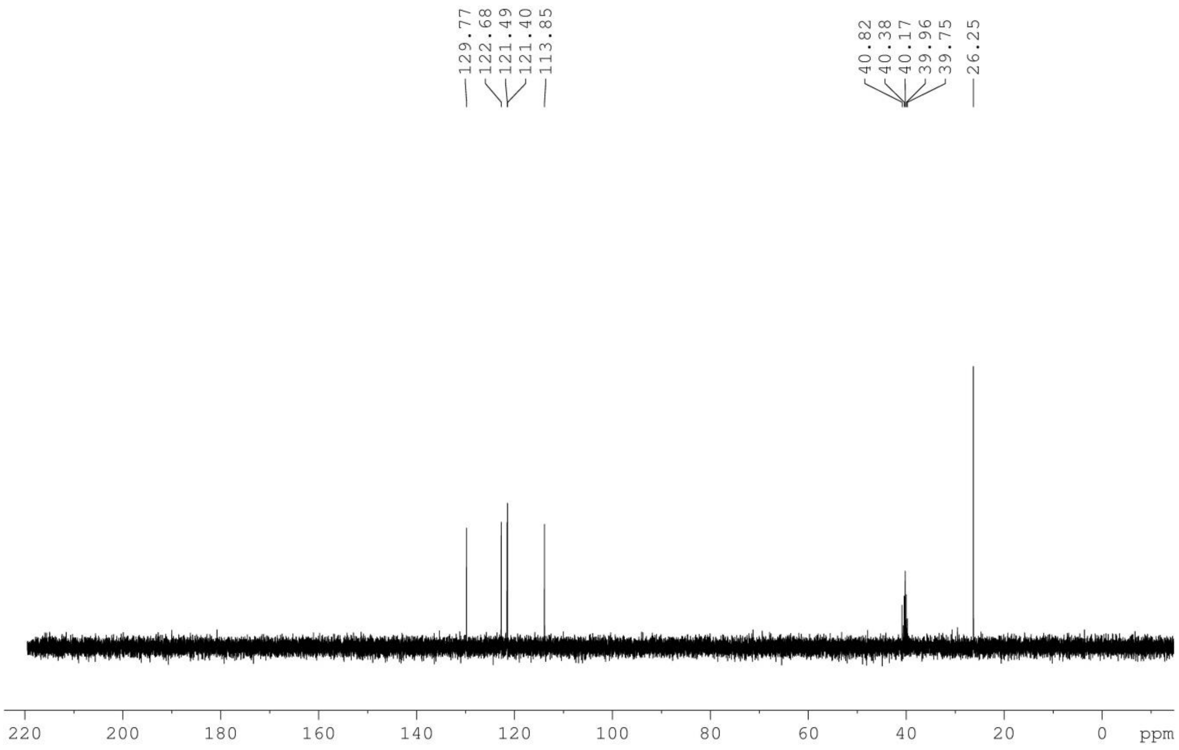

Supplementary Figure S7 DEPT 45 (Y5-P1)

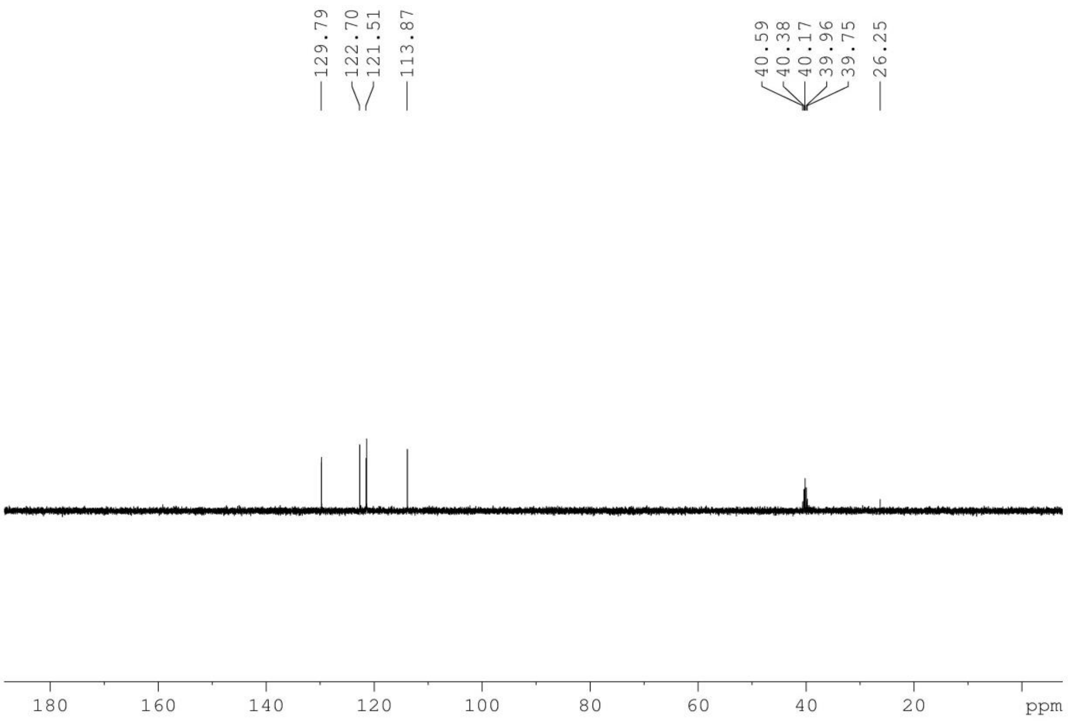

Supplementary Figure S8 DEPT 90 (Y5-P1)

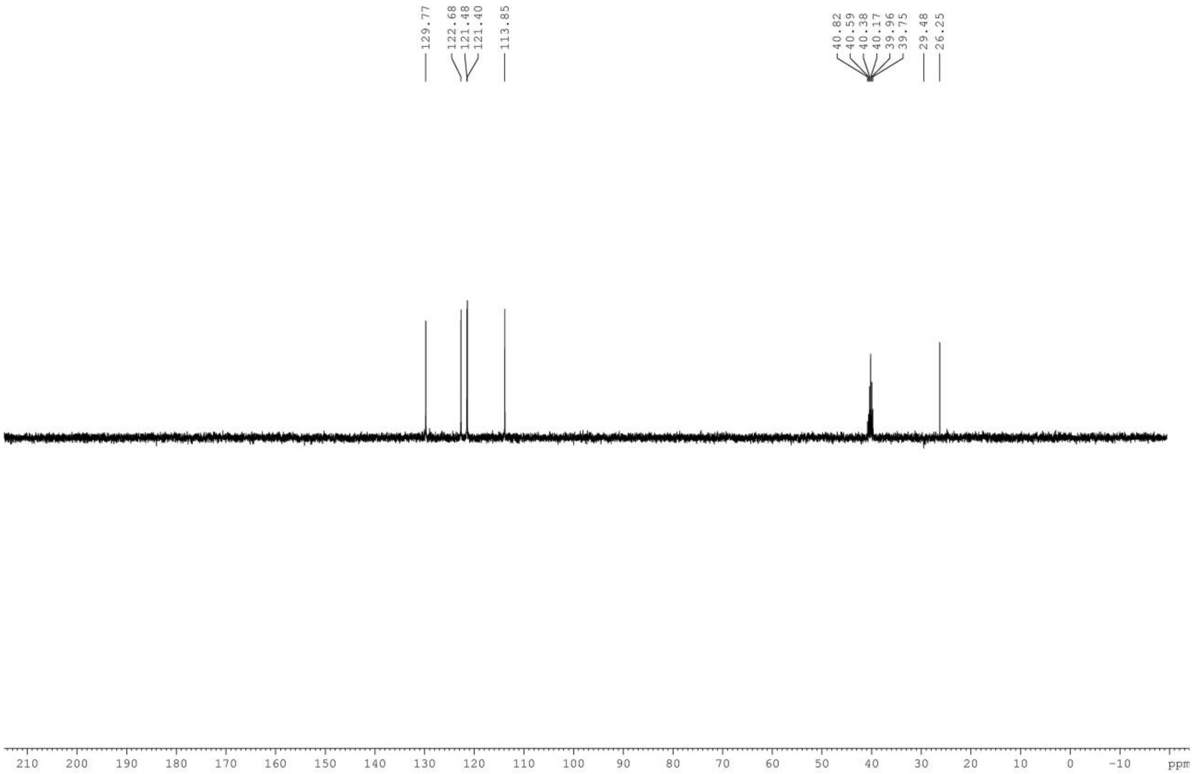

Supplementary Figure S9 DEPT 135 (Y5-P1)

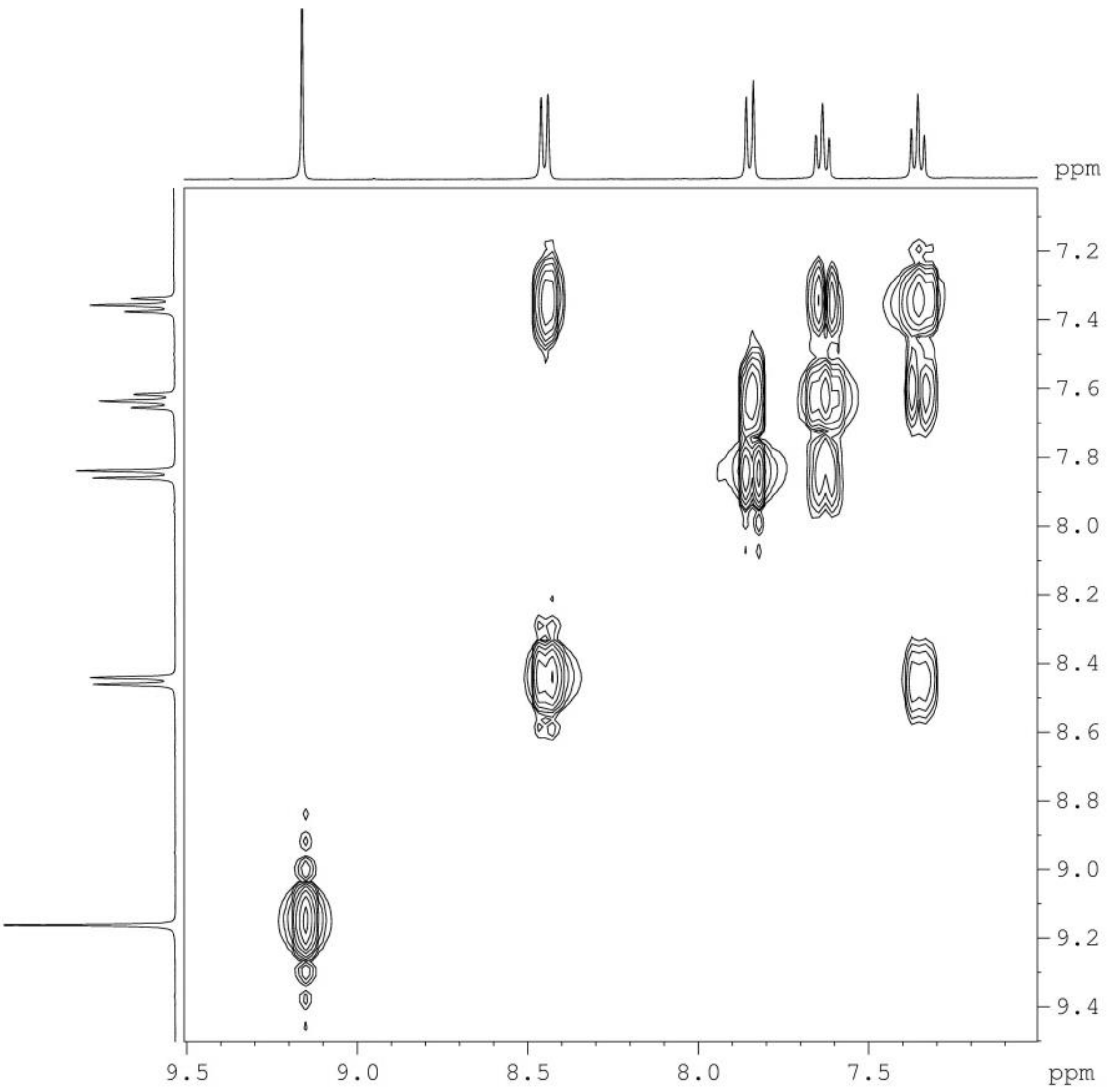

Supplementary Figure S10 ^{1}H- ^{1}H COSY spectrum (Y5-P1)

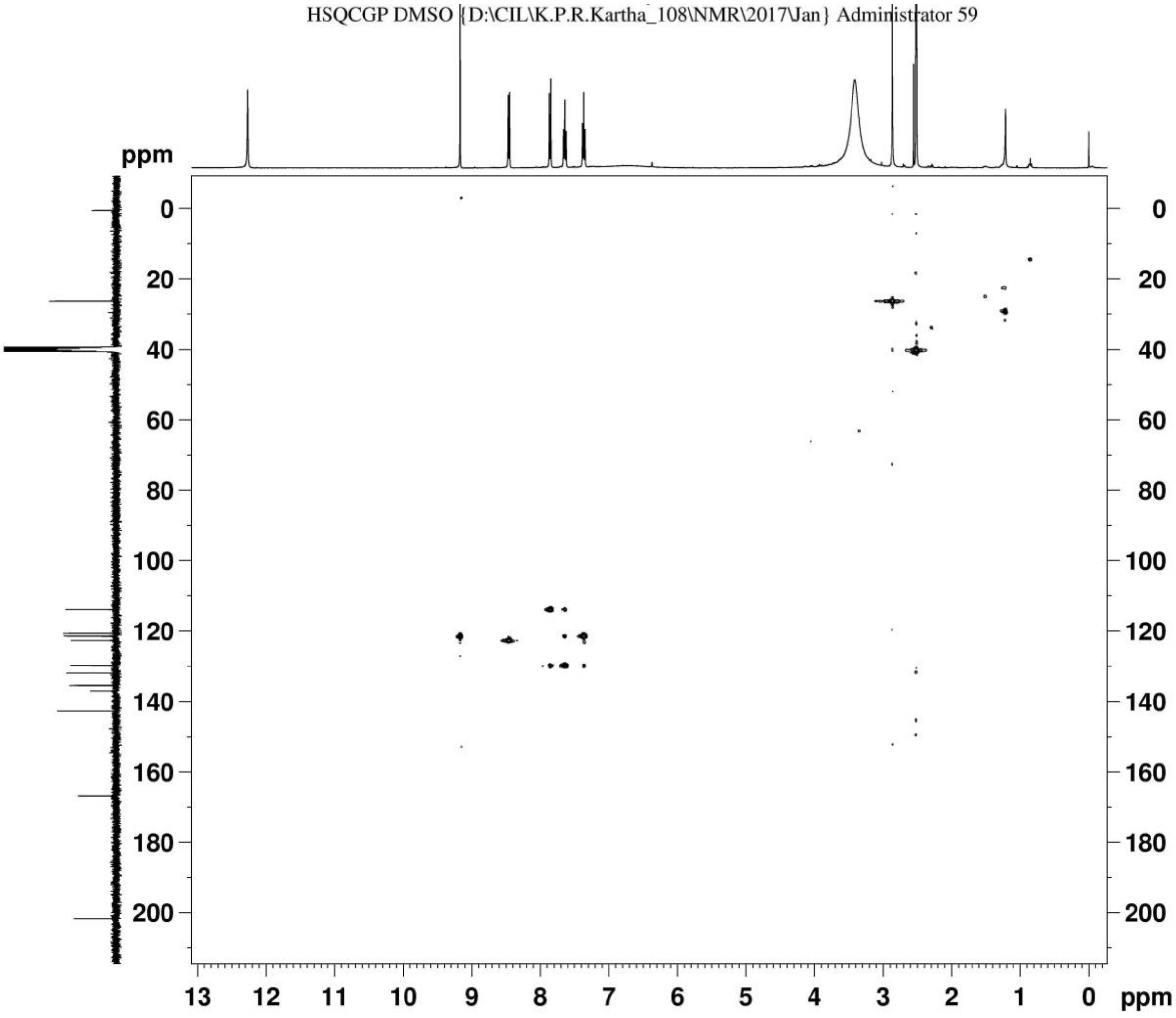

Supplementary Figure S11 ^{1}H- ^{13}C HSQC (Y5-P1)

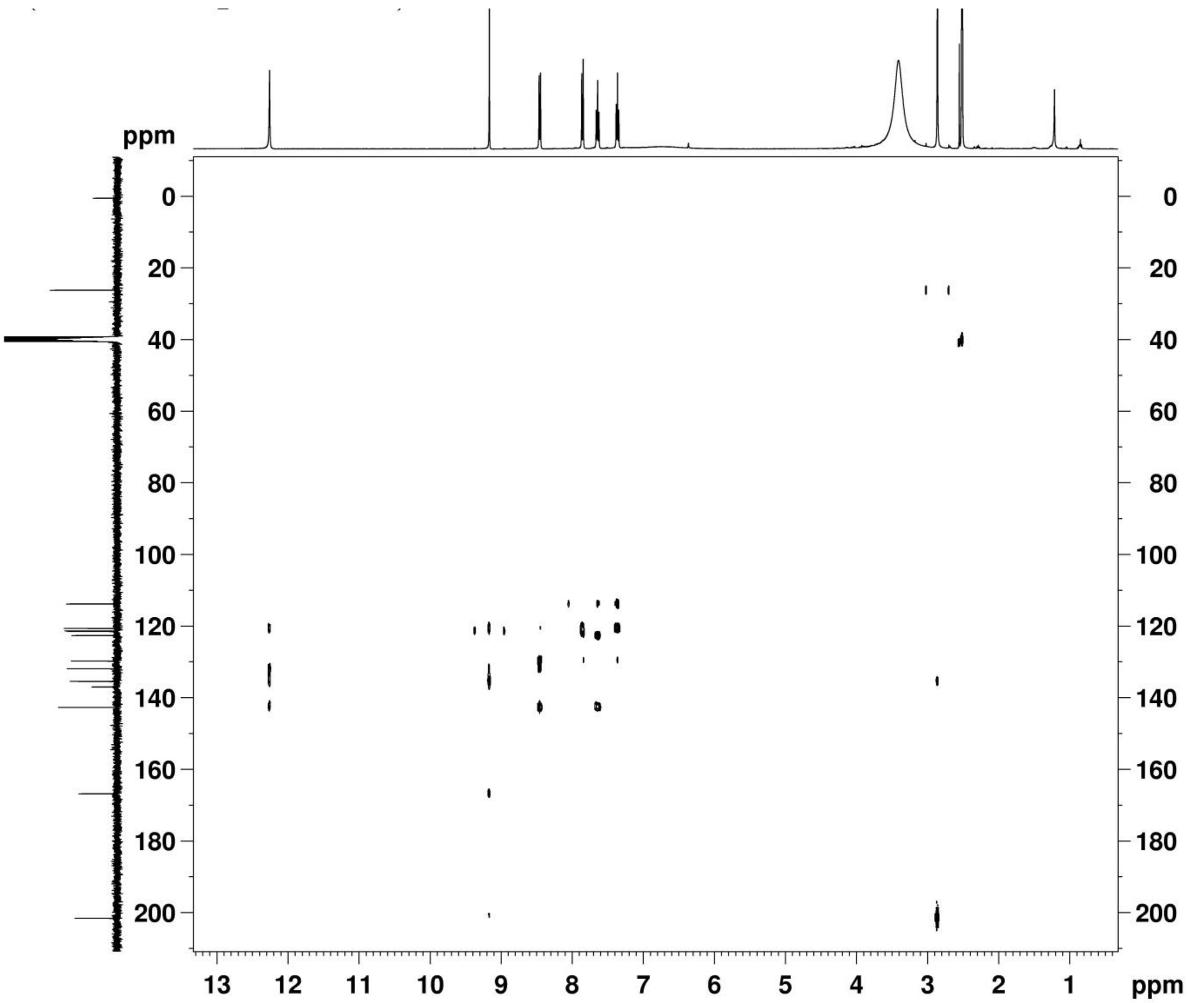

Supplementary Figure S12 ^{1}H- ^{13}C HMBC (Y5-P1)

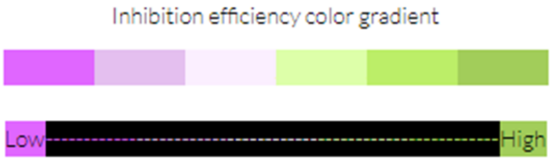

aBiofilm Prediction results

Predicted effeciency of Query Compound	
Query	c12c(cccc1)[nH]c1c2cc(nc1C(=O)C)C(=O)O
Predicted efficiency to target biofilm	High
**Structural information of Query Compound**	
2-D Structure (using JSME editor & mol2ps)	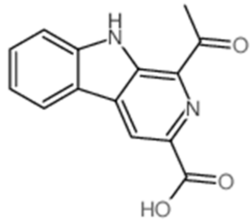
Structural view (using JSME editor & JSmol)	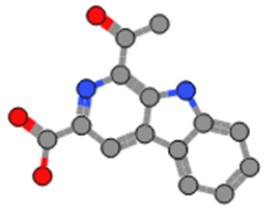
**General Properties of Query Compound**	
Molecular Formula	C14H10N2O3
Formal Charges	0
H-bond acceptors	4
H-bond donors	2
Lipinski acceptors	5
Lipinski donors	2
Rigid bonds	0
Rotatable bonds	2
LogP	2.758
Molecular weight	254.241

Query compound is searched in aBiofilm database and found 0 similar records

Supplementary Figure 13 Prediction of antibiofilm potential of Y5-P1 using *aBiofilm*
